# Focus on ‘Psychiatry and Addiction: A Multi-Faceted Issue’

**DOI:** 10.3390/brainsci15101125

**Published:** 2025-10-20

**Authors:** Fabrizio Schifano, Giovanni Martinotti, Norbert Scherbaum

**Affiliations:** 1 Psychopharmacology, Drug Misuse and Novel Psychoactive Substances Research Unit, University of Hertfordshire Medical School, Hatfield AL10 9AB, UK; giovanni.martinotti@gmail.com; 2 Department of Neuroscience, Imaging, and Clinical Science, “G. d’Annunzio” University of Chieti-Pescara, 66103 Chieti, Italy; 3 LVR-University Hospital Essen, Department of Psychiatry and Psychotherapy, Medical Faculty, University of Duisburg-Essen, 45147 Essen, Germany; norbert.scherbaum@uni-due.de

The reconceptualization of addiction as a neurobiological disease has gained significant traction over recent decades, informed by advances in neuropharmacological and neuroimaging research. Current nosological frameworks provide structured diagnostic criteria that differentiate between substance-related and behavioural (non-substance-related) addictive disorders. Substance use disorders (for general and recent overviews, see [1,2]) encompass classical psychoactive agents (e.g., ethanol, opioids, stimulants), novel psychoactive substances (NPS) [3], and even certain non-psychoactive medications with abuse liability [4]. In contrast, behavioural addictions, e.g., compulsive eating or gambling, remain outside of these frameworks despite converging clinical and neurobiological evidence supporting their inclusion.

At the neuropharmacological level, both substance-related and behavioural addictions are characterized by the dysregulation of meso-corticolimbic dopamine signalling, particularly within the ventral tegmental area (VTA), nucleus accumbens (NAc), and prefrontal cortex (PFC) [5]. These circuits mediate reward salience, motivation, and reinforcement learning, and their perturbation underlies compulsive drug seeking and loss of control [5].

Prevalence data have suggested the frequent co-occurrence of poly-substance use and comorbid behavioural addictions [6] suggesting a shared neuropharmacological vulnerability. Indeed, several pharmacotherapies exhibit anti-craving properties across diagnostic boundaries. For instance, varenicline, a partial nicotinic acetylcholine receptor agonist, reduces craving in both nicotine and alcohol use disorders via the modulation of dopaminergic release in the NAc [7]; bupropion, a norepinephrine-dopamine reuptake inhibitor (NDRI), has demonstrated efficacy in stimulant use disorders and compulsive eating, potentially by enhancing synaptic dopamine in reward-related circuits [8]; naltrexone, a μ-opioid receptor antagonist, reduces alcohol and opioid relapse through the inhibition of opioid-induced dopaminergic activation in the VTA [9]; topiramate, via the modulation of AMPA/kainate receptors and the facilitation of GABAergic transmission, attenuates cue-induced craving in alcohol and cocaine use disorders [10]; and finally, GLP-1 receptor agonists may be effective in treating overeating/obesity, treatment-resistant depression, and both drug (e.g., nicotine; cocaine; possibly opioids) and alcohol use disorders [11]. These examples reflect a transdiagnostic pharmacological profile, arguably supporting the construct of Addiction Spectrum Disorders, a dimensional model recognizing the shared pathophysiological mechanisms underpinning seemingly disparate addictive behaviours.

The high comorbidity between addiction and primary psychiatric disorders further supports the existence of common neuropharmacological substrates. Notably, aberrant salience attribution, which is a core feature of psychotic disorders [12], may arise from dysregulated mesolimbic dopamine activity, mirroring mechanisms observed in addiction. Indeed, antipsychotic-induced D2 receptor antagonism may precipitate a “reward deficiency syndrome”, contributing to the emergence of compensatory addictive behaviour aimed at restoring dopaminergic tone [13,14].

Furthermore, the chronic blockade of D2-like receptors in pancreatic β-cells influences insulin secretion and glucose homeostasis, with antipsychotics possibly disrupting glycaemic regulation and contributing to metabolic syndrome (for an overview, see [15]). Conversely, emerging evidence suggests that glucagon-like peptide-1 receptor agonists (GLP-1 RAs), such as liraglutide and semaglutide, may mitigate antipsychotic-induced weight gain [15]. These agents may also modulate central reward pathways via gut–brain signalling, opening up new avenues for dual-action treatments targeting both metabolic and addictive pathologies [11] (Contribution 1). Importantly, the therapeutic potential of these agents must be weighed against emerging concerns regarding their non-medical use or abuse liability, particularly in populations with heightened reward sensitivity [16].

In line with the above considerations, the sub-topics characterizing this Special Issue (https://www.mdpi.com/journal/brainsci/special_issues/39EC71DF96 (accessed on 3 September 2025) are as follows: alcohol use disorder (AUD); gambling and internet gaming disorder; chemsex; novel antidiabetics/GLP-1 RAs; and stimulant use disorder. We will now introduce each contribution area in more depth.

*Alcohol*

Malewska-Kasprzak (Contribution 2) aimed to summarize experimental and clinical data related to a range of neurotrophins with possible use in alcohol withdrawal and delirium tremens (DT) prediction. According to them, brain-derived neurotrophic factor (BDNF) levels could help in assessing relapse susceptibility, as they are significantly reduced during consumption and gradually increase during abstinence; glial cell line-derived neurotrophic factor (GDNF) may overall influence AUD course through its integral role in the function of dopaminergic neurons; nerve growth factor (NGF) may protect neurons from ethanol-induced cytotoxic damage; neurotrophin 3 (NT-3) levels may be decreased after alcohol exposure; neurotrophin 4 (NT-4) affects chronic alcohol consumption-related oxidative stress; and finally S100B (e.g., a cytosolic calcium-binding protein that is concentrated in astrocytes and released following astroglial injury) may serve as a biomarker for brain damage.

Becciolini et al. (Contribution 3) focused on identifying the best pharmacological treatment for alcohol withdrawal syndrome. In a retrospective case–control study, they assessed the effectiveness and safety of symptom-triggered therapy (STT) compared to fixed-dose regimens (FDRs) in the management of inpatient alcohol detoxification. During a 12-month observation period, a total of 123 patients in the STT group were recruited and compared with 123 adequately matched controls in the FDR group. Overall, STT showed a significantly lower total benzodiazepine dosage (22.50 mg vs. 115.00 mg, *p* < 0.001), a shorter duration of the detoxification phase (48.00 h vs. 201.75 h, *p* < 0.001), and a reduced length of inpatient stay (23.00 days vs. 28.00 days, *p* = 0.003) compared to FDRs. No significant differences in the rates of complications between the two settings were identified.

Bell et al. (Contribution 4) focused on ‘embodied cognition’, which is a recent concept in cognitive science emphasizing the integral role of perception, action, and bodily experience in shaping human thought and understanding. They used the Automated Test of Embodied Cognition (ATEC), providing a comprehensive measure of eight domains of embodied cognition in a 49-subject alcohol use disorder (AUD) sample. Embodied delayed recall was the most frequent impairment (84%), but executive functions (EFs) were also common. Using the ATEC total score, 43% of the sample patients were rated as having a mild or greater level of overall impairment. Using the ATEC total score, younger age, higher education, and a higher premorbid IQ were found to be potential protective factors against cognitive decline. 

Scholl et al. (Contribution 5) focused on a range of clinical-related parameters pertaining to adult children of a parent with an alcohol use disorder (ACoA). They compared ACoA individuals with risky/hazardous alcohol use (*n* = 14) and those not engaged in hazardous use (*n* = 14) with a group of healthy controls through assessing a range of brain structures. They found that both the hazardous and non-hazardous ACoA groups had worse brain ages than the healthy control group (*n* = 100), possibly associated with neuro-developmental differences between the ACoA groups and controls. Furthermore, hazardous ACoA participants had increased predicted epigenetic age difference scores compared to both the control group (*n* = 34) and the non-hazardous ACoA participant group.

Whilst alcohol dependence is associated with several neuropsychological abnormalities, such as increased impulsivity or attentional bias towards drug-related stimuli, it is unclear whether these abnormalities decline after long-term abstinence from alcohol. Hence, Rabl et al. (Contribution 6) focused on 130 alcohol-dependent patients consecutively admitted for a duration of 14–26 weeks to an inpatient rehabilitation treatment centre. Investigations were conducted at entry, after six weeks, and during the final two weeks of their inpatient treatment. The investigators found that there was a significant decrease in patients’ scores over time for both craving (*p* < 0.001) and impulsivity (*p* < 0.001), but no significant changes in their attentional bias or inhibitory control scores. They concluded that patients’ risk of post-discharge relapse may remain high.

*Internet/gaming disorder*

According to Varchetta et al. (Contribution 7) internet addiction (IA) and related behaviour, such as Internet Gaming Disorder (IGD) and social media addiction (SMA), may be characterized by gender differences. Hence, they carried out a 585-participant cross-sectional study. Males exhibited significantly higher scores for IA and IGD, while females showed higher scores for SMA and some dimensions of ‘phubbing’ (phone snubbing, i.e., focusing on their phone whilst in the presence of significant others). The authors suggest that tailored interventions should address unique online behaviours and emotional regulation challenges in males and females.

Attention deficit hyperactivity disorder (ADHD) and autism spectrum disorder (ASD) have been related to increased risk for behavioural addictions including online gaming. Hence, the aim of Simonelli et al. (Contribution 8) was to address this topic by exploring the prevalence of IGD in a consecutive sample of young people with ASD and with ADHD, compared with a normal control group. Some 72% of the ADHD sample were above the IGD cut-off, compared with 45% in the ASD group and 9.5% in the NC group. ASD patients with IGD presented with greater severity and more severe attention problems, whilst ASD patients with IGD were the most clinically severe group.

According to Orsolini et al. (Contribution 9), a new “Modern-Type Depression” (MTD) clinical syndrome is currently being observed. As a consequence, their aim was to identify the prevalence of MTD in a sample of 543 young Italian videogame players (aged 18–35) by providing a clinical characterization of MTD within a group of IGD individuals (IGD+) versus a group without IGD (IGD−). Subjects were administered the 22-item Tarumi’s Modern-Type Depression Trait Scale (TACS-22), the Motives for Online Gaming Questionnaire (MOGQ), and the IGD Scale-Short Form (IGDS9-SF). Within the IGD sample, around 34% of subjects had MTD. Higher scores in the TACS-22 were positively predicted based on the total score for IGDS9-SF (*p* = 0.003), the MOGQ “Escape from Reality” subscale (*p* = 0.014), and MOGQ “Fantasy” (*p* = 0.011) and were negatively predicted based on the MOGQ “Competition” subscale (*p* = 0.035) [F (4538) = 17.265; *p* < 0.001]. They concluded that MTD may show a strong association with IGD.

Tagliaferri et al. (Contribution 10) carried out a 28-study systematic review to assess the role of trait boredom in digital behavioural addictions. Overall, it was found that trait boredom may serve as a mediator and moderator in the relationship between depression, alexithymia, and dysfunctional digital behaviours. Hence, they concluded that boredom may represent a central psychological element in better understanding behavioural addictions.

*Chemsex*

With the help of an anonymous, cross-sectional, online survey, Gertzen et al. (Contribution 11) assessed whether there were differences in certain psychosocial aspects between chemsex participants and non-participants and which factors led to negative impacts from the chemsex experience in these subjects. They collected a total of 3257 datasets from 107 chemsex participants and found that values for shame proneness, more negative aspects of queer identity, sexual anxiety, intravenous substance use, and having had a difficult process coming out were all significant predictors of feeling negative impacts.

*GLP-1 RAs*

Since glucagon-like peptide-1 (GLP-1) is involved in a range of central and peripheral pathways related to appetitive behavior, with the help of a mixed-methods approach to analyze online discussions, Arillotta et al. (Contribution 1) explored the effects of GLP-1 receptor agonists (GLP-1 RAs) on substance and behavioral addictions. A total of 5859 Reddit threads and related comments were extracted. In total, 29.75% of alcohol-related comments, 22.22% of caffeine-related comments, and 23.08% of nicotine-related comments clearly stated a cessation of the intake of these substances following the start of GLP-1 RA prescription. Conversely, mixed results were found for cannabis intake, and only limited, anecdotal data were available for cocaine, entactogen, and dissociative drug misuse. Regarding behavioral addictions, 21.35% of comments reported a compulsive shopping interruption, whilst sexual drive/libido elements reportedly increased in several users.

*Stimulant misuse*

Cue exposure therapy, which specifically targets the psychological and physiological responses elicited by drug-related cues, is a promising treatment approach in cocaine use disorder. Hence, Brobbin et al. (Contribution 12) systematically reviewed and categorized the range of cocaine cues used in research. Some 235 articles were included in this analysis; the cues identified included images, paraphernalia, drug-related words, the smell of cocaine, auditory stimuli presented via audiotapes, video recordings, scripts, and virtual reality environments, often combining multiple modalities; sample sizes ranged from a single case study to reports including 1974 participants. It was suggested that further research should focus on enhancing cue exposure techniques by incorporating more immersive and personalized stimuli.

*Conclusions*

As highlighted here, AUD-related contributions to this Special Issue focus on a range of topics, including the identification of biomarkers that can possibly predict the emergence of delirium tremens; the optimal use of benzodiazepines in treating withdrawal; related impairments to mental functioning whilst considering the embodied cognition approach; neuro-anatomical differences between children of a parent with an alcohol use disorder and control groups; and finally the possible persistence of a range of neurobiological abnormalities following long-term inpatient alcohol detoxification (Contribution 6). The papers on gambling and internet gaming disorders emphasize the role of gender differences in internet and social media use and misuse, as well as the determinant roles of both autistic spectrum disorders and ADHD in characterizing and facilitating the emergence of internet gaming disorders. Finally, important conclusions are reached with regard to certain psychological elements characterizing chemsex enthusiasts, the different cues used to elicit cocaine cravings in experimental studies, and, finally, the potential role of novel antidiabetics/GLP-1 RAs in shaping the future of addiction psychopharmacology.
